# Hybrid Transition Metal Dichalcogenide/Graphene Microspheres for Hydrogen Evolution Reaction

**DOI:** 10.3390/nano10122376

**Published:** 2020-11-28

**Authors:** Marco Lunardon, JiaJia Ran, Dario Mosconi, Carla Marega, Zhanhua Wang, Hesheng Xia, Stefano Agnoli, Gaetano Granozzi

**Affiliations:** 1 Department of Chemical Sciences, University of Padova, Via F. Marzolo 1, 35131 Padova, Italy; marco.lunardon.5@phd.unipd.it (M.L.); jiajia.ran@studenti.unipd.it (J.R.); dario.mosconi@unipd.it (D.M.); carla.marega@unipd.it (C.M.); stefano.agnoli@unipd.it (S.A.); 2 State Key Laboratory of Polymer Materials Engineering, Polymer Research Institute, Sichuan University, Chengdu 610065, China; zhwangpoly@163.com (Z.W.); xiahs@scu.edu.cn (H.X.)

**Keywords:** reduced Graphene Oxide Aerogel Microspheres, prGOAM, 3D hybrid system, electrospray, ice templating, TMDC, HER

## Abstract

A peculiar 3D graphene-based architecture, i.e., partial reduced-Graphene Oxide Aerogel Microspheres (prGOAM), having a dandelion-like morphology with divergent microchannels to implement innovative electrocatalysts for the hydrogen evolution reaction (HER) is investigated in this paper. prGOAM was used as a scaffold to incorporate exfoliated transition metals dichalcogenide (TMDC) nanosheets, and the final hybrid materials have been tested for HER and photo-enhanced HER. The aim was to create a hybrid material where electronic contacts among the two pristine materials are established in a 3D architecture, which might increase the final HER activity while maintaining accessible the TMDC catalytic sites. The adopted bottom-up approach, based on combining electrospraying with freeze-casting techniques, successfully provides a route to prepare TMDC/prGOAM hybrid systems where the dandelion-like morphology is retained. Interestingly, the microspherical morphology is also maintained in the tested electrode and after the electrocatalytic experiments, as demonstrated by scanning electron microscopy images. Comparing the HER activity of the TMDC/prGOAM hybrid systems with that of TMDC/partially reduced-Graphene Oxide (prGO) and TMDC/Vulcan was evidenced in the role of the divergent microchannels present in the 3D architecture. HER photoelectron catalytic (PEC) tests have been carried out and demonstrated an interesting increase in HER performance.

## 1. Introduction

In the last decade, materials characterized by pores of different size and shapes have received considerable attention from both the academic and industrial world. The porous structure endows such materials with a high surface area and, more importantly, allows a subtle control over mass transport thanks to size exclusion and size dependent permeation. This behavior is of crucial importance for separation, adsorption, gas storage and catalysis, that are key processes involved in different fields, ranging from biology to environmental protection [1,2] and from chemistry to energy conversion and storage [3,4,5].

Porous materials based on carbon are particularly attractive. The low density, the high strength per weight and the ability to bind with various atoms through different hybrid orbitals (sp^n^) make carbon an extraordinarily versatile building block for the construction of porous structures with intriguing morphologies and multifunctional properties. Graphene (G) has been the most studied carbon allotrope of the last fifteen years. This interest is due to the G peculiar physicochemical properties (electronic and thermal conductivity, mechanical and chemical stability) and to its 2D structure, which results in a very high intrinsic surface area [6,7,8]. The specific theoretical surface area of a single G sheet is 2630 m^2^/g [9], but due to the strong tendency to aggregation, experimental values are generally far below this value. The rational assembly of G based 3D architectures allows preserving the 2D G patches while maximizing the accessible surface area, resulting in a system with high porosity [10]. Among the various G based 3D architectures, G aerogels combine the exceptional intrinsic G properties with the specific properties of aerogels (lightness, low dielectric permittivity, etc.) [11,12,13,14]. In recent years, they have been extensively studied, and the synthesis strategies can be divided into three categories: chemical vapor deposition (CVD) [15,16], self-assembly [17,18,19] and direct assembly via a template [20,21,22].

**Figure 1:**
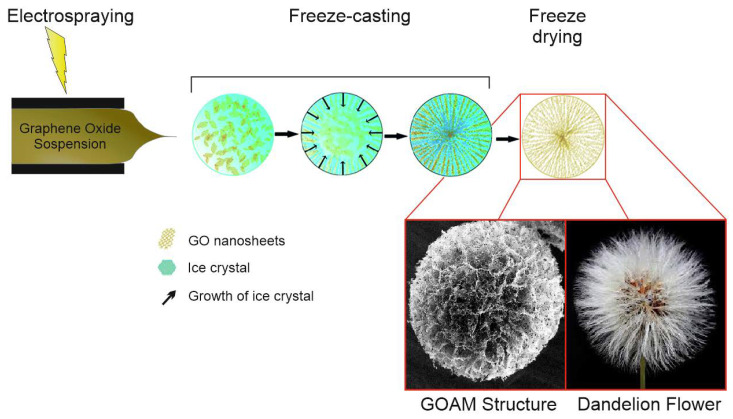
Schematic representation of Graphene Oxide Aerogel Microspheres (GOAMs) synthesis and the final structure of the microspheres showing a kind of resemblance with the *dandelion* (Taraxacum) flowers.

Recently, some of us have reported the synthesis of graphene oxide (GO) aerogel microspheres (GOAMs) [23], i.e., a 3D porous structure with microchannel central divergence, by combining electrospray [24,25] with freeze-casting techniques [26] (Figure 1). In these 3D architectures, the interconnected G sheets arranged radially forms open ended spheres with a radius of ~100 µm, resembling *dandelion* flowers. Interestingly, thermal treatment in inert atmosphere [27] can produce partial reduced GO aerogel microspheres (prGOAM). These materials, given their highly porous and hydrophobic structure, have excellent adsorption capacity for various organic solvents and oils, finding applications in environmental pollutant treatment and water purification [23].

The coupling of electrospray and freeze casting allows for a high production speed per single unit (single GOAM sphere). By approximating the droplet radius of the starting dispersion with that of the final GOAM structure (~100 µm) and considering a nebulization speed of 0.1 mL/min, a production speed of ~400 units per second is obtained. With this production speed, assuming that the GOAMs structures are arranged on a plane, e.g., a compact hexagonal pattern, the obtained surface coverage speed is ~8 cm^2^/min. These values refer to a single electrospray nozzle; therefore, the implementation with a microfabricated nozzle multiplex matrix would increase the production speed in proportion to the number of nozzles [28,29]. From this, it follows that this technique can quickly produce a significant amount of a porous carbon material.

Due to its properties and versatility, G is also used in the production of high-performance electrocatalysts for the hydrogen evolution reaction (HER) [30]. Among these, transition metals dichalcogenides (TMDCs)/G hybrid systems are widely studied as alternatives to Pt-based electrocatalysts for HER [31,32,33]. In this work, we report on the preparation of an innovative hybrid electrocatalyst coupling TMDCs nanosheets with *dandelion-like* prGOAMs (TMDC/prGOAM) by electrospray and freeze casting techniques. The aim is to prove that it is possible to integrate into the prGOAM graphene network the functional properties of catalysts without altering the microchannel central divergence morphology. The procedure of incorporation has been optimized using exfoliated MoSe_2_, and subsequently it was successfully extended to other exfoliated TMDCs, i.e., MoS_2_, WS_2_ and WSe_2_.

The new TMDC/prGOAM catalysts display an interesting improvement of the HER activity in an acidic environment compared to the pristine TMDC, confirming a kind of synergetic interaction between the TMDC catalyst and the graphene layers. Moreover, the comparison of the HER results of TMDC/prGOAM modified electrodes with those obtained from TMDC/prGO and TMDC/Vulcan analogous hybrid materials (benchmark samples) allowed us to demonstrate the role of the 3D architecture in the catalytic HER performance. More interestingly, this 3D architecture is also preserved after the electrochemical tests. The hybrid electrocatalysts were also HER verified under light in photoelectron catalytic (PEC) tests displaying interesting results.

## 2. Materials and Methods

### 2.1. Synthesis of Graphene Oxide Aerogel Microspheres (GOAMs)

GOAMs were prepared following a modified version of the procedure by Liao et al. [23]. A 6 mg/mL GO aqueous suspension was prepared, stirred overnight and then transferred into a syringe with a curved needle. The electrospray parameters were as it follows: the flow was set to 0.1 mL/min; the electrospray voltage: 10 kV; the distance between the tip of the syringe and the surface of the collection beaker: 10 cm. The beaker (electrically grounded) containing n-hexane was cooled to −78 °C by an acetone-dry ice bath.

When the nebulized cone encounters the cold receiving solution, the spherical water microdroplets quickly freeze converting into GO ice microspheres, which were recovered through a metallic sieve and transferred into a cooled vial to avoid the thaw of the sample. Finally, the GOAMs were obtained by removing the water by freeze-drying. It is worth noting that the n-hexane level in the collection beaker significantly decreases for long electrospray-synthesis time (>1 h). Since the distance between the syringe tip and the solution meniscus affects the dimensions of the microstructure, the variation in time of this distance can influence the average size of the microspheres [23].

### 2.2. Preparation of Exfoliated MX_2_ TMDCs (M = Mo, W; X = S, Se)

Exfoliated MX_2_ TMDCs nanosheets were obtained from the following bulk materials: MoS_2_, MoSe_2_, WS_2_ and WSe_2_. The exfoliation procedure was carried out by Li-intercalation according to a standard protocol [34,35,36,37,38]. In a dry-box, 2 mmol of TMDC (average size of 60–90 µm) and 5 mmol of LiBH_4_ were ground together in a mortar and transferred into a tube. The tube was then connected to a Schlenk line and after three vacuum-N_2_ cycles, it was left under N_2_ atmosphere and heated into a sand bath at 330 °C for 4 days. The resulting solid Li_x_MX_2_ was poured into 300 mL of Milli-Q water, previously saturated by N_2_ bubbling, and the so obtained black suspension was sonicated for 30 min. To remove the generated LiOH, three centrifugal washing cycles were performed (10,000 rpm, RCF 19236-*g*, 15 min); at the end of each cycle, the supernatant was replaced with 50 mL of degassed Milli-Q water. Finally, the clean TMDC solid was dispersed 100 mL of Milli-Q water and freeze-dried to get a solid powder.

### 2.3. Synthesis of MX_2_/GOAM and MX_2_/prGOAM

The TMDC/GOAM samples were obtained by the addition of a defined amount of exfoliated TMDC to the starting GO aqueous solution and following the same procedure described above. The TMDC concentration was optimized in the case of MoSe_2_, by HER characterizing the final materials: the best electrochemical activity was obtained by using 5 mg/mL (0.02 M). For the successive preparation of the MoS_2_/GOAM, WS_2_/GOAM and WSe_2_/GOAM, the mass concentration was kept constant.

The TMDC/prGOAM samples were obtained from the corresponding TMDC/GOAM systems, after heat treatment in a tubular oven at 450 °C (ramp 5 °C min^−1^) for 2 h in an Ar:H_2_ = 90:10 (100 sccm in total) atmosphere. Two control experiments were performed in the same conditions at 150 and 900 °C.

The TMDC:C ratio in the nebulized drops during the electrospray step is assumed the same as that of the starting suspension. Therefore, the TMDC:C ratio in TMDC/GOAM and TMDC/prGOAM systems should not deviate significantly from the nominal one of the initial suspensions.

### 2.4. Preparation of Benchmark Samples

The electrocatalytic performances of the MoSe_2_/prGOAM systems have been compared with some benchmarks based on MoSe_2_/carbon systems: GO and commercial Vulcan XC-72 (Cabot Corporation, Boston, MA, USA). The MoSe_2_/carbon were obtained according to the following procedure: 6 mg of reference carbon material and 5 mg of MoSe_2_ were ground together in a mortar and the obtained mixture was heated at 450 °C in a tubular furnace with a Ar:H_2_ = 90:10 (100 sccm in total) flow for 2 h.

A similar argument to what has been said for the preparation of TMDC/GOAM and TMD/prGOAM is also valid for the benchmark sample TMDC: C ratio.

### 2.5. Physico-Chemical Characterization

Scanning electron microscopy (SEM) micrographs were acquired using a field emission source equipped with a GEMINI column (Zeiss Supra VP35) with an acceleration voltage of 5 kV using secondary electron detection. Energy Dispersive X-ray Analysis (EDX) chemical mapping was recorded on the same instrument using an Oxford Instruments detector. Raman spectra were obtained with a ThermoFisher DXR Raman microscope. The spectra were recorded using a laser with an excitation wavelength of 532 nm (0.1 mW), focused on the sample with a 50× objective (Olympus).

X-ray photoemission spectroscopy (XPS) data were acquired by a custom-designed UHV system equipped with an EA 125 Omicron electron analyser with five channeltrons, working at a base pressure of 10^−10^ mbar. Core level photoemission spectra were taken in normal emission using the Mg K_α_ emission line (hv = 1253.6 eV) of a nonmonochromated dual-anode DAR400 X-ray source. High resolution spectra were acquired using 0.5 s dwell time, 0.1 eV energy steps, and 20 eV pass energy. The multipeak analysis of the C 1s, Mo 3d, W 4f, Se 3d and S 2p photoemission lines was performed by means of Voigt functions and subtracting a Shirley background using the *KolXPD* software [38].

### 2.6. Electrochemical Characterization

The electrocatalytic studies were performed in a Teflon electrochemical cell (see in the Supplemental Information file, Figure S1b), using a Ag/AgCl (3M KCl) electrode (calibrated as +0.218 V vs. the reversible hydrogen electrode, RHE) and a glassy carbon (GC) rod as reference (RE) and counter electrode (CE), respectively. The working electrode (WE) was prepared by depositing 25 µL of the catalyst ink on a GC electrode (area delimited to 4.5 mm diameter), corresponding to an active material loading of 142 µg cm^−2^. The catalyst ink was formulated by dispersing 10 mg of sample and 20 μL of Nafion in 1 mL of ethanol, then drop casted on the WE, and finally dried in vacuum. To deposit undamaged GOAMs, it was necessary to widen the hole of the micropipette tip. Due to the typical GOAMs’ size (~100 µm) it was rather difficult to create a mechanically stable and homogeneous layer of uniform thickness on the WE: to achieve the goal the just drop-casted layer was added with 50 μL of ethanol. The HER measurements were carried out in N_2_-saturated 0.5 M H_2_SO_4_ solution at room temperature. Polarization curves were recorded from +0.05 V to −0.30 V vs. RHE using a scan rate of 0.005 V s^−1^. Polarization curves with the same parameters were recorded in the presence of white LED (light intensity equal to 97 mW cm^−2^, emission spectrum is shown in Figure S1a) for PEC measurements. Cyclic voltammetry (CV) curves under light exposure were recorded from +0.05 V to −0.15 V vs. RHE using a scan rate of 0.005 V s^−1^ to investigate the photodegradation process of MoSe_2_/prGOAM. The distance between the LED and the WE in electrolyte was about 2 cm. Due to the equilibrium potential for HER, the overpotential values (η) reported below are equal to the electrochemical potential in absolute value.

The potential scan was carried employing a potentiostat Autolab PGSTAT204 (Metrohm). The potentiostat unit was paired to the optical desk to control the LED during PEC measurements. Currents presented in the text are normalized by the geometrical area and iR-corrected by using the resistance determined by electrochemical impedance spectroscopy (EIS) measurements. EIS was performed at η = 0.28 V (100 kHz to 0.1 Hz) and fitted using a R (RQ) as equivalent circuit.

## 3. Results and Discussion

### 3.1. Chemical and Structural Characterization

The first step of this study was to test the feasibility to make hybrid TMDC/GOAM systems that preserve the *dandelion-like* morphology. For the realization of these systems, we have chosen to follow a bottom-up approach. Few-monolayer TMDC and GO were used as building blocks for the target 3D architecture. The search for the optimal TMDC:GO ratio was carried out on the MoSe_2_/GOAM system taking into consideration both the maintenance of the 3D *dandelion-like* architecture and the HER performance. The preparation of the other TMDC/GOAM systems was subsequently adopted for the same optimized ratio value obtained on MoSe_2_/GOAM.

**Figure 2:**
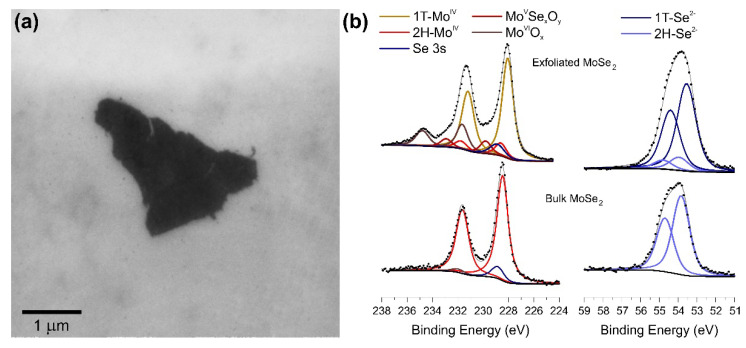
(**a**) Typical SEM image of the exfoliated MoSe_2_ supported on a Au substrate; (**b**) XPS characterization of exfoliated and bulk MoSe_2_. On the left the Mo 3d region, on the right the Se 3d region.

The exfoliated MoSe_2_ nanosheets were obtained as described in the Section 2 and characterized by SEM, Raman and XPS, comparing the data with bulk material ones. Chemical exfoliation produces few layer thick nanosheets that according to the SEM images (Figure 2a), have an average lateral size in the micrometer range.

The exfoliated TMDCs samples are typically composed of a mixture of 1T and 2H polymorphs, which can be identified by Raman spectroscopy: in Figure S2 we report the Raman spectra of the bulk MoSe_2_ and of the exfoliated sheets. The Raman spectrum of bulk MoSe_2_ shows three signals at 167, 241 and 285 cm^−1^, which can be associated, respectively, with the E_1g_, A_1g_ and E_2g_ normal modes of the 2H-MoSe_2_. After exfoliation, the normal modes E_1g_ and A_1g_ of the 2H phase (171 and 238 cm^−1)^, and the modes J_1_, J_2_, J_3_ and A_1g_ of the 1T phase (108, 154, 228 and 287 cm^−1^) become visible [39]. The Raman spectrum of the exfoliated sample reported in Figure S2b is compatible with the literature data of few-layers MoSe_2_ [40] and show the typical feature of both polymorphs.

An alternative and more quantitative evaluation of the exfoliated material was obtained using XPS (Figure 2b), which allows calculating the 2H:1T ratio and the amount of oxide formed as a consequence of air exposure (see Tables S1 and S2 for details). The Mo 3d region of the MoSe_2_ bulk material can be deconvoluted in two doublets and a single peak at a binding energy (BE) of about 229 eV corresponding to the Se 3s signal. The main doublet, with the Mo 3d_5/2_ and 3d_3/2_ peaks centered at 228.5 eV and 231.6 eV, is attributed to Mo (IV) species of 2H-MoSe_2_; the high BE doublet (232.0–234.2 eV) is relative to molybdenum (VI) oxide deriving from surface oxidation [41,42]. As mentioned above, the intercalation of Li ions induces the 2H→1T conversion, which causes the appearance of 1T-MoSe_2_ signals in the spectra of the exfoliated sample. In the Mo 3d photoemission line, a doublet with peaks centered at 228 eV and 231.2 eV corresponding to the Mo (IV) 3d_5/2_ and 3d_3/2_ and a peak at 228.6 eV relative to the Se 3s have to be included in the fitting procedure [43]. The analysis of the Se 3d core level is analogous to that of Mo 3d; for the bulk MoSe_2_ material only one doublet with peaks centered at 53.8 eV and 54.7 eV is identified, which is associated with Se^2−^ ions of 2H-MoSe_2_, while for the exfoliated sample, a second doublet at lower BE (53.5 and 54.4 eV) corresponding to the 1T-MoSe_2_ [43] phase is observed. In Table S1 we report the 2H:1T ratio determined by XPS. It is possible to quantify the degree of the 2H→1T transformation (final 88% of 1T), with only a limited oxidation of the material.

Different samples of MoSe_2_/GOAM were prepared, changing the concentration of exfoliated MoSe_2_ in the initial suspension while keeping the GO concentration constant (6 mg/mL). The samples were later characterized by SEM (Figure S3) and by EDX chemical mapping (Figure S4). The 3, 5, 7 and 9 mg/mL MoSe_2_ concentrations were tested to find the TMDC:GO ratio range that preserves the *dandelion-like* morphology. The morphological GOAM features are conserved until to 7 mg/mL MoSe_2_ concentration.

Outside this range, a Taylor cone instability, and the absence of microstructure in the final material were observed. In detail, using the 9 mg/mL MoSe_2_ concentrations, aggregates of irregular shapes and different sizes were observed (Figure S3d).

As for the actual mechanism of the formation of the hybrid materials, we can propose that during the freeze-casting step, the expanding ice crystals create contact zones between the GO sheets and the exfoliated TMDC, promoting their interaction through van der Waals forces. If enough carboxyl groups (from GO) make substantial crosslinking between the GO sheets, during the subsequent lyophilisation, when the ice compression relaxes, the established crosslinking points allow preserving the central diverging microchannel structure. Therefore, when a high TMDC concentration is reached, the formation of sufficient crosslinking points might be inhibited.

**Figure 3:**
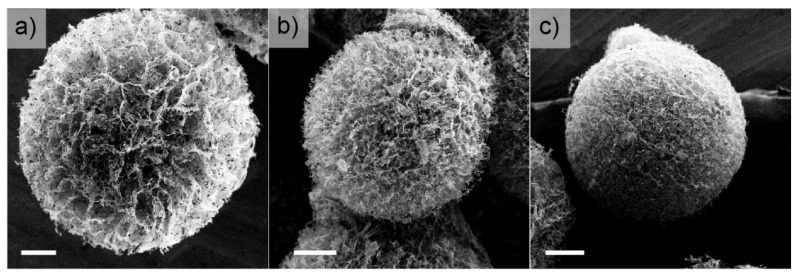
SEM images of MoSe_2_/prGOAM samples reduced to 450 °C in the atmosphere Ar:H_2_ = 90:10 (100 sccm in total). MoSe_2_/prGOAM samples obtained using a 3 (**a**), 5 (**b**), and 7 mg/mL (**c**) concentration of MoSe_2_. Scale bars are 50 µm.

**Figure 4:**
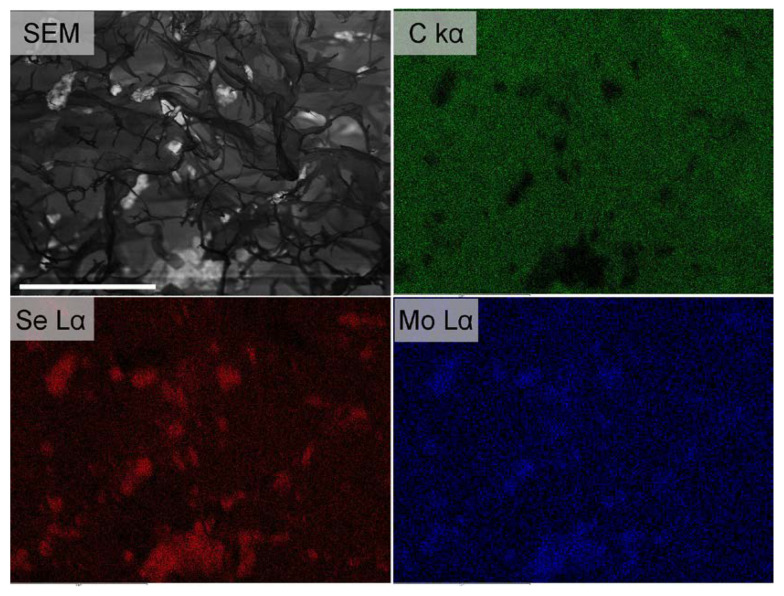
SEM images and EDX chemical map of the 5 mg/mL MoSe_2_/prGOAM. EDX showing the element’s maps of C (green) and Mo (blue) and Se (red) signals. Scale bars are 20 µm.

However, the as-prepared MoSe_2_/GOAM systems are not suitable for electrochemical investigations since they are mechanically unstable, and the microstructure is completely lost when the samples are dispersed in polar solvents (water, ethanol, DMF). These solvents compete with the weak interactions (H-bonds) of the crosslinking points in the GOAM structure, inducing the separation of the GO sheets. Thence, the obtained TMDC/GOAM samples were annealed to stabilize the GOAM structure, causing a covalent crosslinking between graphene sheets [44,45], while partially reducing the GO to prGO. We explored the best annealing temperature in the case of MoSe_2_/rGOAM samples by using a tubular oven in an Ar:H_2_ = 90:10 flow (100 sccm in total) for 2 h at different temperatures (150, 450, 900 °C). The new MoSe_2_/prGOAM materials allowed preparing a stable catalyst ink to test their HER activities (vide infra). In addition, the heat treatment induces a partial reduction of GO to prGO, so partially healing the graphene structure and restoring the electrical conductivity [27,46]. The XPS and Raman spectra were used to characterize the annealing process at 450 °C: the increase of C*sp*^x^/CO_x_ peaks area ratio in XPS spectra (Figure S5 and Table S3), and the decrease in the D/G and D’/G peaks intensity ratio in the Raman graphene signals (see Figure S6 and Table S4) confirms the partial establishment of the sp^2^ network following the 450 °C annealing [47]. Additionally, the analysis of the second-order Raman data of graphene suggests the presence of 2–3 layers of graphene-related material; this value is invariant to the annealing process [48]. Interestingly, the SEM micrographs in Figure 3 and Figure S7 show that the annealing preserves the *dandelion-like* morphology. The EDX chemical maps in Figure 4 show the dispersion of the exfoliated MoSe_2_ sheets in the rGOAMs: actually, it is possible to identify features of about 1 µm, compatible with exfoliated MoSe_2_, which decorate the GO sheets, most probably through plane-to-plane interactions. Figure S8 shows an optical microscope image of a section of a single MoSe_2_/prGOAM included in the epoxy resin; this image further proves that the presence of TMDC does not alter the formation of centrally diverging microchannels.

Finally, the thermal treatment induces the 1T- to 2H-MoSe_2_ transition [49] (see Raman in Figure S2), thus replacing the metallic phase, which typically shows better electrocatalytic performances, with the semiconducting polymorph, which on the other hand can exhibit photoactivity (vide infra) [43].

### 3.2. Electrocatalytic HER Characterization

The MoSe_2_/prGOAM systems can be dispersed in ethanol without losing their *dandelion-like* morphology, and even after the electrocatalytic test, no substantial differences could be observed (see Figure S9).

**Figure 5:**
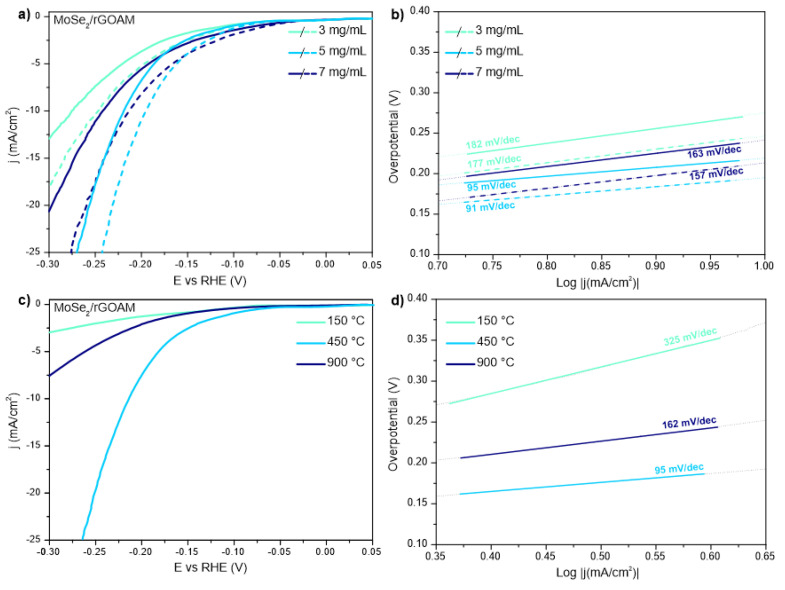
Polarization curves (**a**) and Tafel plots (**b**) for hydrogen evolution reaction (HER) of MoSe_2_/prGOAM samples annealed at 450 °C obtained by using different concentrations of MoSe_2_. Solid lines represent experiments in dark conditions, dashed lines the PEC-HER measurements performed under illumination. Polarization curves (**c**) and Tafel plot (**d**) for HER of MoSe_2_/prGOAM samples obtained with 5 mg/mL MoSe_2_ and different annealing temperatures.

Figure 5a shows the polarization curves of the MoSe_2_/prGOAM systems obtained using different concentrations of MoSe_2_, after annealing at 450 °C; being the MoSe_2_ nanosheet mostly present as 2H polymorph, we monitored PEC behavior as well, using a white LED to illuminate the working electrode (see Experimental Section). As for performance descriptors, we report the Tafel slopes and the overpotentials at 10 mA cm^−2^ (η_10_). The activity parameters obtained from the polarization curves are shown in Table 1. The sample obtained using a MoSe_2_ concentration of 5 mg/mL shows the best performances. Under illumination, all samples show an improvement of η_10_ of about 11% and a gain in terms of current density (+j_10_) ranging from +3 to +7 mA/cm^2^; the +j_10_ value was obtained considering the j increase under light vs. dark at the value of η_10_ recorded in the dark condition. This result could find an explanation in the reminder of what has been said for the mechanical stability of MoSe_2_/GOAMs. The concentration of MoSe_2_ included in the carbon structure influences the number of crosslinking points in the graphene network. A lower interconnection between the graphene sheets limits the possible electrons percolation paths in the hybrid system. Therefore, an excessive concentration limits the movement of electrons while a low concentration disadvantages the number of available catalytic sites. Based on the significant improvement under light, we retained that also a stability test was useful to understand if any photodegradation process of the catalyst is occurring. In detail, further polarization curves were acquired after 1000 CVs under light exposure (Figure S10 and Table S4). An η_10_ shift of 5 and 9 mV respectively for the light and dark condition and a decrease of +j_10_ value of 1.3 mA/cm^2^ are registered. The material used as WE was also characterized by Raman after the EC test (Figure S11 and Table S5); no significant variation was observed concerning the already discussed MoSe_2_/prGOAM spectra (Figures S2 and S6). These results confirm a substantial stability under light exposure, proving that no significant photodegradation process occurs for MoSe_2_/prGOAM.

After finding the optimal MoSe_2_ concentration, we also explored the role of the different annealing temperatures (150, 450, 900 °C) (Figure 5c): the results displayed definitely give the best performances after a 450 °C annealing (remind that the SEM images do not show any change in the morphology at different T, Figure 3 and Figure S9). According to the literature, the electronic conductivity of the reduced GO depends significantly on the annealing temperature, passing from 400 S cm^−1^ after annealing at 900 °C to values lower than 50 S cm^−1^ at temperatures below 450 °C [27,46]. On the other hand, 200 °C sets the threshold for the 1T→2H transition in exfoliated MoSe_2_ [49]. Therefore, 450 °C might represent a compromise between the transformation from GO to prGO and polymorphic transformation of MoSe_2_. On the other hand, higher temperatures may also induce defects healing, which can be counterproductive in electrocatalysis [46,49,50,51]. The tested temperatures allow investigating the limit cases illustrated above: at 150 °C, 1T-MoSe_2_ should be preserved, but the electronic conductivity of the GO should be relatively low, while at 900 °C, the opposite situation should occur.

In addition to η_10_ values, also the Tafel slopes for temperatures different from 450 °C (see Figure 5d) give an important feedback: they are not attributable to the typical mechanisms for HER (see the electrochemical data in Table 2). The sample reduced at 150 °C shows the worst performances, with a maximum current density (at −0.4 V vs. RHE) lower than the 10 mA/cm^2^ and with a Tafel slope greater than 300 mV/dec. This behavior is mainly attributable to the low electronic conductivity of GO sheets treated at low temperatures [27]. On the other hand, the sample reduced at 900 °C shows η_10_ values of about 330 mV and a Tafel slope of about 160 mV/dec. The poor performance is probably due to the strong prGO hydrophobicity, which limits the permeation of the electrolyte into microchannels. On the other hand, at 450 °C, the electronic conductivity and hydrophobicity of the prGO are sufficiently balanced, and the corresponding MoSe_2_/prGOAM sample shows a η_10_ value of 220 mV and a Tafel slope of 95 mV/dec. This value suggests a Volmer-Heyrovsky mechanism for the HER with the adsorption process as rate determining step.

**Figure 6:**
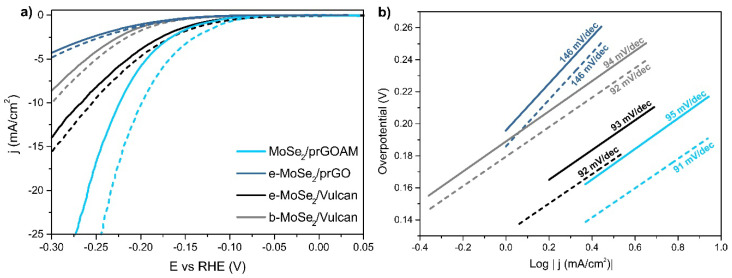
(**a**) Polarization curves for MoSe_2_/prGOAM and the benchmark systems (see text). The dashed curves were obtained by illuminating the working electrode with white light (see Experimental Section); (**b**) related Tafel plots.

To understand if the *dandelion-like* morphology brings some advantage to the electrocatalytic performance, we compared the MoSe_2_/prGOAM with some benchmark materials. MoSe_2_/C systems where C is either GO and commercial Vulcan XC-72 have been prepared, as described in the Experimental Section, using either exfoliated MoSe_2_ (e-MoSe_2_) or bulk MoSe_2_ (b-MoSe_2_) as the active material and using the same annealing conditions as in the optimized MoSe_2_/prGOAM sample. The tested benchmark samples are e-MoSe_2_/prGO, e-MoSe_2_/Vulcan and b-MoSe_2_/Vulcan. The polarization curves of the different samples are shown in Figure 6, and the related electrochemical data are summarized in Table 3.

The e-MoSe_2_/prGO and MoSe_2_/prGOAM systems differ by their preparation procedures: in the former, the hybrid system is realized by grinding and therefore, the relative final structure is a simple arrangement of 2D units without a particular order; in the latter, instead, the sheets of MoSe_2_ and those of GO are arranged inside a 3D spherical microstructure (Figure 4), forming channels where the electrolyte can permeate. Figure 6a and Table 3 show that the 3D structure leads to a significant increase in the electrocatalytic performance.

Figure 6 compares the results of MoSe_2_/prGOAM with those obtained for both e-MoSe_2_/Vulcan and b-MoSe_2_/Vulcan systems. Vulcan has two types of porosity, the mesopores (2–50 nm) for the dispersion of the catalysts and the macropores (>50 nm) for the diffusion of reagents and products, designed to optimize electrocatalytic performance in systems such as fuel cells [52]. The MoSe_2_/prGOAM system shows better performance with a gain of about 50 mV in η_10_ compared to the MoSe_2_/Vulcan system. In terms of the Tafel slope (Figure 6b), the three systems show similar values (about 90 mV/dec), confirming a Volmer-Heyrovsky mechanism. More differences are observed in terms of exchange current density (j_0_): the MoSe_2_/prGOAM system shows a value of j_0_ approximately double than that of the e-MoSe_2_/Vulcan and about 5-times compared to that of the b-MoSe_2_/Vulcan.

Considering the PEC behavior (see dashed curves in Figure 6), more significant improvements in performance can be noted for the MoSe_2_/prGOAM system; in particular, an improvement of η_10_ of about 25 mV and a gain in terms of current density (+j_10_) of 6.6 mA/cm^2^ are observed, which are superior to all the other systems with the same photoactive component (i.e., MoSe_2_) but different structure and morphology of the carbon support. The best performance in PEC can be read as an indirect proof of a greater light-harvesting by MoSe_2_ inside the GOAM architecture.

After optimization of the MoSe_2_/prGOAM preparation procedures, we extended our investigation to a series of MX_2_/prGOAM systems where M = Mo, W; X = S, Se. In the SI, we report a section where an extended characterization of the exfoliated TMDCs is reported (Figures S12–S17 and Tables S6–S11).

The same TMDC mass loading (5 mg/mL) was explored to test the different TMDC/prGOAM systems, without any optimization for the different MX_2_. For this reason, the reported data must be considered as explorative. From SEM images and relative EDX data, no relevant changes are observed in the whole MX_2_/prGOAM series (Figure S18).

**Figure 7:**
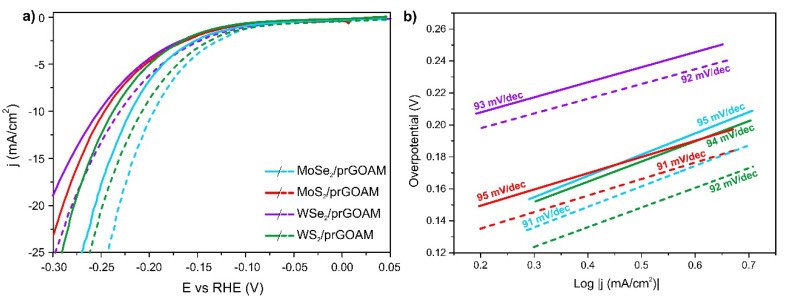
(**a**) Polarization curves for HER and PEC-HER activities of TMDC/prGOAM systems. The dashed curves were obtained by illuminating the working electrode with white light. (**b**) Tafel plots for electrochemical tests of different TMDCs/prGOAM hybrids.

The polarization curves of the TMDC/prGOAM samples are shown in Figure 7, and the relative electrochemical data are reported in Table 4. The reported trend is in agreement with the literature (MoSe_2_ > WS_2_ > MoS_2_ > WSe_2_) [53]. Most probably the reported trend is due to different HER active sites in the different TMDCs. The primary active sites for MoSe_2_ and WS_2_ are both the metal and the chalcogen edges. On the other hand, the active sites for MoS_2_ and WSe_2_ are only Mo and Se edges, respectively [53,54]. However, it must be noted that, with proper optimization, the performances of the other TMDC/prGOAM hybrids could be enhanced, but this goes beyond the purposes of this work.

In kinetics terms, however, WX_2_/prGOAM shows the best performances, with an approximately double value of j_0_ compared to MoX_2_/prGOAM. Furthermore, considering the PEC behavior, the WS_2_/prGOAM is the system with the most remarkable light enhancement, with a reduction of η_10_ value of about 30 mV, a current density gain (+j_10_) of about 7 mA/cm^2^, and an exchange current increase of about 65 μA/cm^2^. The WSe_2_/prGOAM system shows a worse performance concerning WS_2_ with a η_10_ value of about 250 mV and a Tafel slope higher than 130 mV/dec. The high Tafel slope is probably to be attributed to the high oxidation degree of exfoliated WSe_2_ (about 50%, see XPS data in Table S10).

## 4. Conclusions

In this work, we demonstrated that a bottom-up approach based on the use of electrospray and freeze casting techniques starting from GO and exfoliated TMDCs is capable to provide hybrid 3D TMDC/GOAM architectures without significantly altering the *dandelion-like* microchannel arrangement with central divergence typical of the GOAM. We have used MoSe_2_ as benchmark material to optimize many process parameters. Then we have applied the protocol to a wide gamut of other TMDCs materials (i.e., MoS_2_, MoSe_2_, WS_2_ and WSe_2_). Moreover, such TMDC/GOAM structure can be easily transformed after a thermal annealing at an intermediate temperature (450 °C) into a mechanically ore stable one, i.e., TMDC/prGOAM still retaining the same architecture. After annealing, covalent crosslinking is established while the electronic conductivity of the sample is increased because of the partial reduction of the GO sheets. Together with the finally tuned wettability of the sample, the annealing procedure makes the TMDC/prGOAM hybrid systems suitable for electrocatalytic experiments: stable electrodes can be prepared and tested. As a consequence of the actual HER screening, we found that the MoSe_2_/prGOAM sample provides the best HER performances.

To explore the role of the *dandelion-like* architecture, we have also compared the HER results of MoSe_2_/prGOAM with those of related benchmark samples, i.e., hybrid MoSe_2_/carbon systems where the *dandelion-like* architecture is not present. Our tests pinpoint better HER performances for the sample with the 3D architecture with respect to the chosen benchmarks. For the sake of honesty, it should be noted that TMDCs/G-based systems with better HER performances with respect to our TMDCs/prGOAM samples are reported in the literature [55,56]. However, a full optimization of our 3D catalysts was not carried out, since the main goal of this study was the check of the feasibility of preparing HER working electrodes using complex 3D hybrid architectures. Future work is certainly needed for a full optimization of the electrodes by a fine tuning of the graphene/TMDC interactions inside the *dandelion-like* microspheres.

## Figures and Tables

**Table 1:** HER activity parameters of MoSe_2_/prGOAM from data reported in Figure 5a.

MoSe_2_ Conc.	η_10_ (mV)	η_10_ LED (mV)	+j_10_ (mA/cm^2^)	Tafel Slope (mV/dec)	Tafel Slope LED (mV/dec)
3 mg/mL	276 (5)	247 (5)	3.6	182 (1)	177(1)
5 mg/mL	220 (5)	194 (5)	6.6	95 (2)	91 (1)
7 mg/mL	241 (5)	215 (5)	4.4	163 (1)	157 (1)

**Table 2:** HER activity parameters of MoSe_2_/prGOAM from data reported in Figure 5c.

Annealing T (°C)	η_10_ (mV)	Tafel Slope (mV/dec)
150	-	325(1)
450	220 (5)	95 (2)
900	332 (5)	162(1)

**Table 3:** HER and PEC-HER activity parameters from data reported in Figure 6.

	η_10_ (mV)	Tafel Slope (mV/dec)	η_10_ LED (mV)	Tafel Slope LED (mV/dec)	+j_10_ (mA/cm^2^)	j_0_ (μA/cm^2^)	j_0_ LED (μA/cm^2^)
e-MoSe_2_/prGO	393 (5)	146 (2)	382 (5)	146 (2)	0.8	46 (3)	53 (3)
b-MoSe_2_/Vulcan	314 (5)	92 (1)	299 (5)	92 (1)	1.5	10 (1)	11 (1)
e-MoSe_2_/Vulcan	266 (5)	93 (1)	254 (5)	92 (1)	1.5	27 (4)	37 (3)
MoSe_2_/prGOAM	220 (5)	95 (1)	194 (5)	91 (1)	6.6	47 (2)	73 (4)

**Table 4:** Electrochemical data from LSV shown in Figure 7.

	η_10_ (mV)	Tafel Slope (mV/dec)	η_10_ LED (mV)	Tafel Slope LED (mV/Dec)	+j_10_ (mA/cm^2^)	j_0_ (μA/cm^2^)	j_0_ LED (μA/cm^2^)
MoS_2_/prGOAM	246 (5)	95 (1)	230 (5)	91 (2)	3.1	32 (2)	61 (2)
MoSe_2_/prGOAM	220 (5)	95 (1)	194 (5)	91 (1)	6.6	47 (2)	73 (4)
WS_2_/prGOAM	237 (5)	94 (2)	208 (5)	92 (1)	6.8	85 (9)	139 (3)
WSe_2_/prGOAM	253 (5)	92 (1)	231 (5)	92 (1)	3.7	81 (6)	174 (4)

## Supplementary Materials

The following are available online at https://www.mdpi.com/2079-4991/10/12/2376/s1, Figure S1. (a) Emission spectra of white LED and (b) Teflon electrochemical cell with quartz windows for PEC. Figure S2. Raman spectra of bulk MoSe_2_ (a), exfoliated MoSe_2_ (b), MoSe_2_/GOAM hybrid (c) and MoSe_2_/prGOAM hybrid samples (d). Figure S3. (a–c) SEM images of MoSe_2_/GOAM samples, obtained using respectively the 3, 5, 7 and 9 mg/mL solution of MoSe_2_. Scale bars are 50 µm. Figure S4. SEM images and EDX chemical map of the 5 mg/mL MoSe_2_/GOAM sample, showing the overlap of C (red) and Mo (green) signals. Scale bars are 50 µm. Figure S5. XPS spectra of the C 1 s region of GO (left) and of the MoSe_2_/prGOAM sample reduced to 450 °C (right). Figure S6. Deconvolution of the Raman spectrum of graphene oxide of MoSe_2_/GOAM and MoSe_2_/prGOAM reduced to 450 °C (right). Figure S7. SEM images of MoSe_2_/prGOAM samples obtained by reduction at 150 °C (left) and 900 °C (right). Scale bars are 100 µm. Figure S8. Optical microscope image (63×) of MoSe_2_/prGOAM samples obtained by reduction at 450 °C with different scale bars. Figure S9 Comparison of the SEM images of the MoSe_2_/prGOAM sample before and after the electrochemical tests. The reference sample is that obtained by heat treatment at 450 °C using a 5 mg/mL solution of MoSe_2_. Scale bar are 50 µm. Figure S10. Polarization curves (a) and Tafel plots (b) for HER of as-prepared MoSe_2_/prGOAM samples annealed at 450 °C (black) and after 1000 CVs under light exposure (red). Solid lines represent experiments in dark conditions, dashed lines the PEC-HER measurements performed under illumination. Figure S11. Raman spectra of graphene oxide and MoSe_2_ of MoSe_2_/GOAM and MoSe_2_/prGOAM reduced to 450 °C (right) after 1000 CVs under light exposure. Figure S12. Raman spectra of bulk (a) exfoliated (b) and prGOAM hybrid (c) MoS_2_ samples. Figure S13. XPS spectra of Mo 3d (left) and S 2p (right) regions for exfoliated and bulk MoS_2_ samples. Figure S14. Raman spectra of bulk (a) exfoliated (b) and prGOAM hybrid (c) WS_2_ samples. Figure S15. XPS spectra of W 4f (left) and S 2p (right) regions for exfoliated and bulk WS_2_ samples. Figure S16. Raman spectra of bulk (a) exfoliated (b) and prGOAM hybrid (c) WSe_2_ samples. Figure S17. XPS spectra of W 4f (left) and Se 3d (right) regions for exfoliated and bulk WSe_2_ samples. Figure S18. SEM images and related EDX maps of the samples (a,b) MoS_2_/prGOAM, (c,d) WS_2_/prGOAM and (e,f) WSe_2_/prGOAM. In EDX maps the green signal correspond to C, while red to Mo or W. Scale bars are 50 µm for SEM images and 20 µm for EDX maps. Table S1. Binding Energy (BE) values and composition of Mo 3d core level of the XPS data shown in Figure 2b of the main text. The reported BE values error is ±0.1 eV. Percentage value of nonoxidized Mo and 2H: 1T ratio of MoSe_2_ are reported as well. Table S2. Se 3d BE values and surface composition of the XPS data shown in Figure 2b of the main text shown in Figure 2b. Se 3s BE values are reported as well. The BE values error is ±0.1 eV. Table S3. BE values and composition of C 1s photoemission line. The reported values error is ±0.1 eV. Table S4. HER activity parameters of MoSe_2_/prGOAM from data reported in Figure S10. Table S5. Fitting parameters calculated for MoSe_2_/GOAM and MoSe_2_/prGOAM in the Raman spectra in Figures S6 and S11. Table S6. BE values and composition of Mo 3d core level of MoS_2_ samples. The reported BE values error is ±0.1 eV. Percentage value of nonoxidized Mo and 1T:2H ratio of MoS_2_ are reported as well. Table S7. BE values and composition of S 2p region of MoS_2_ samples. S 2s BE values are reported as well. The error on BE values is ±0.1 eV. Table S8. BE values and composition of W 4f core level of WS_2_ samples. The reported BE values error is ±0.1 eV. BE of W 5p_3/2_, percentage value of nonoxidized W and 1T:2H ratio of WS_2_ are reported as well. Table S9. BE values and composition of S 2p region of WS_2_ materials. BE values error is ±0.1 eV. Table S10. BE values and composition of W 4f core level of WSe_2_ samples. The reported BE values error is ±0.1 eV. BE of W 5p_3/2_, percentage value of nonoxidized W and 1T:2H ratio of WSe_2_ are reported as well. Table S11. BE values and composition of Se 3d region of WSe_2_ materials. BE values error is ±0.1 eV.
