# Supplementary material for: Hybrid Transition Metal Dichalcogenide/Graphene Microspheres for Hydrogen Evolution Reaction
Source: Nanomaterials (Basel). 2020 Nov 28;10(12):2376. doi: 10.3390/nano10122376 (PMC7759811; doi:10.3390/nano10122376)
Supplement: Supplementary file 1 [file nanomaterials-10-02376-s001.pdf]

nanomaterials

Supplementary Materials

Hybrid transition metal dichalcogenide/graphene microspheres for hydrogen evolution reaction

Marco Lunardon [1], JiaJia Ran [1], Dario Mosconi [1], Carla Marega [1], Zhanhua Wang [2], Hesheng Xia [2], Stefano Agnoli [1] and Gaetano Granozzi [1,*]

[1] Department of Chemical Sciences, University of Padova, Via F. Marzolo 1, 35131 Padova, Italy; marco.lunardon.5@phd.unipd.it (M.L.); jiajia.ran@studenti.unipd.it (J.R.); dario.mosconi@unipd.it (D.M.); carla.marega@unipd.it (C.M.); stefano.agnoli@unipd.it (S.A.)

[2] State Key Laboratory of Polymer Materials Engineering, Polymer Research Institute, Sichuan University, Chengdu 610065, China; zhwangpoly@163.com (Z.W.); xiahs@scu.edu.cn (H.X.)

* Correspondence: gaetano.granozzi@unipd.it; Tel.: +39-3347151920

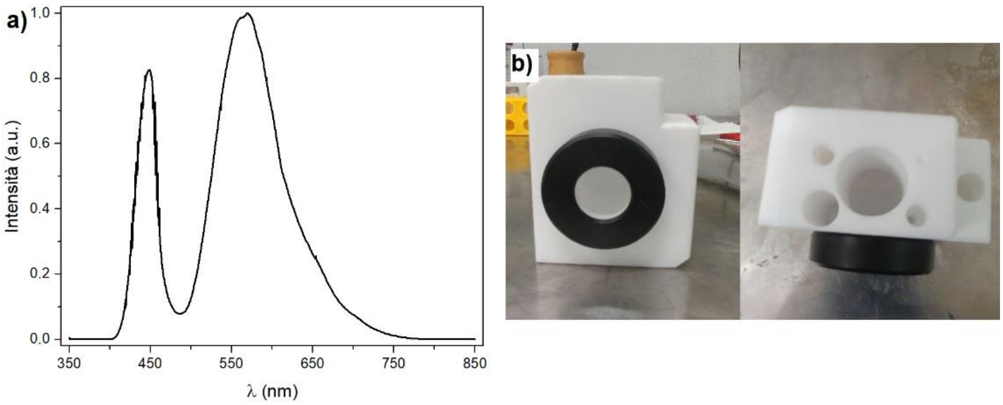

Figure S1. a)Emission spectra of white LED and b)teflon electrochemical cell with quartz windows for PEC.

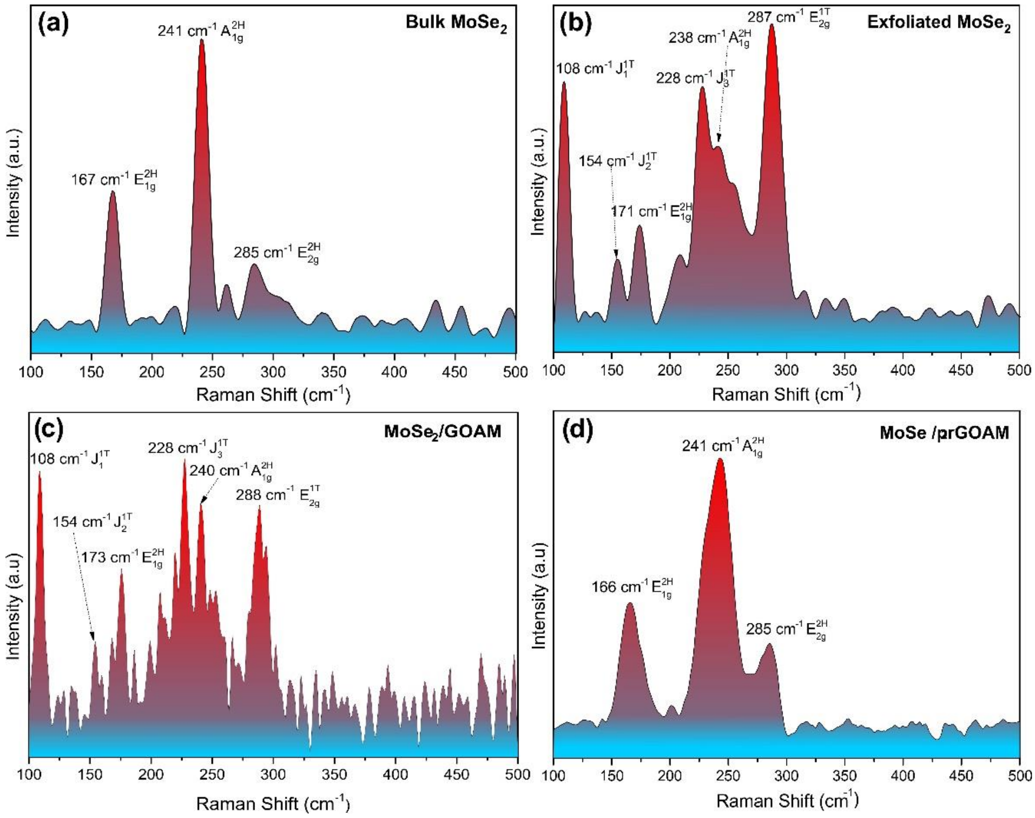

Figure S2. Raman spectra of bulk $MoSe_2$ (a), exfoliated $MoSe_2$ (b), $MoSe_2$/GOAM hybrid (c) and $MoSe_2$/prGOAM hybrid samples (d).

Table S1. Binding Energy (BE) values and composition of Mo 3d core level of the XPS data shown in Figure 2b of the main text. The reported BE values error is ± 0.1 eV. Percentage value of non-oxidized Mo and 2H: 1T ratio of $MoSe_2$ are reported as well.

	**Exfoliated $MoSe_2$**		**Bulk $MoSe_2$**	
Mo 3d	*BE (eV)*	*%*	*BE (eV)*	*%*
1T-Mo^{IV}	228.0	66.0	-	-
2H-Mo^{IV}	228.6	8.6	228.5	97.0
$Mo^{V}Se_xO_y$	229.8	8.4		
$Mo^{VI}O_x$	231.8	17.0	232.0	3.0
1T:2H	88:12		0:100	
Mo(IV)/Mo	75%		97%	

Table S2. Se 3d BE values and surface composition of the XPS data shown in Figure 2b of the main text shown in Figure 2b. Se 3s BE values are reported as well. The BE values error is ± 0.1 eV.

	**Exfoliated $MoSe_2$**		**Bulk $MoSe_2$**	
Se 3d	*BE (eV)*	*%*	*BE (eV)*	*%*
1T-Se^{2-}	53.5	86.9	-	-
2H-Se^{2-}	53.9	13.1	53.8	100
2H-Se 3s	228.8	-	229.2	-

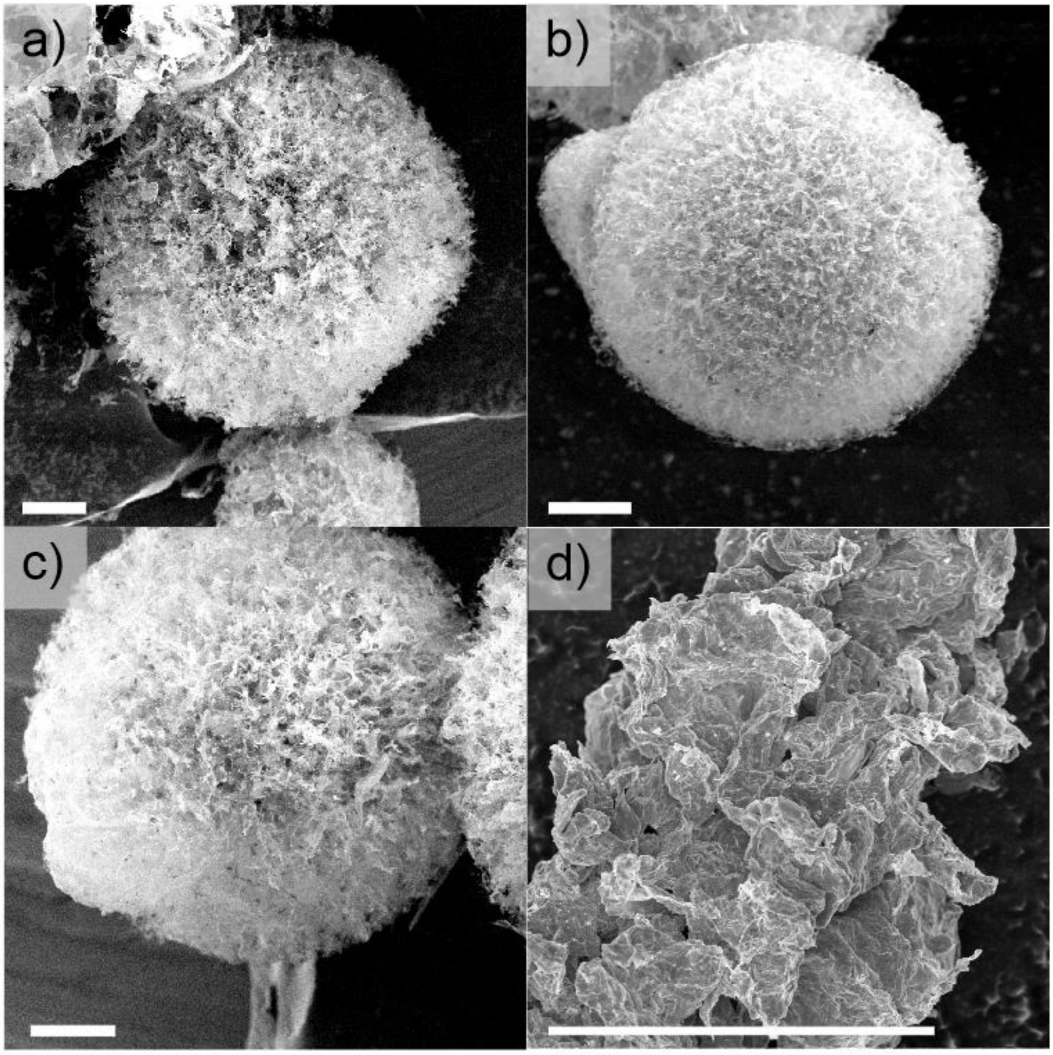

Figure S3. a-c) SEM images of $MoSe_2$/GOAM samples, obtained using respectively the 3, 5, 7, and 9 mg/mL solution of $MoSe_2$. Scale bars are 50 μm.

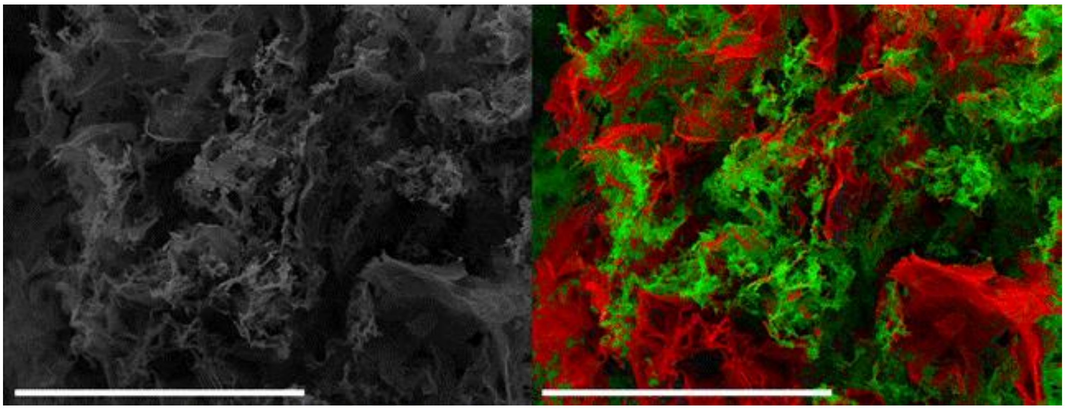

Figure S4. SEM and EDX chemical map of the 5 mg/mL $MoSe_2$/GOAM sample, showing the overlap of C (red) and Mo (green) signals. Scale bars are 50 μm.

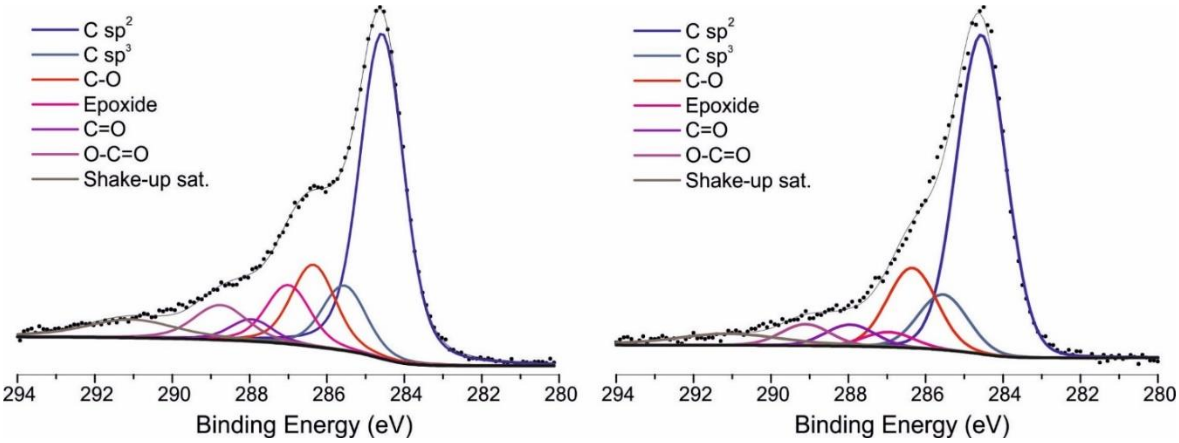

Figure S5. XPS spectra of the C 1s region of GO (left) and of the $MoSe_2$/prGOAM sample reduced to 450 °C (right).

Table S3. BE values and composition of C 1s photoemission line. The reported values error is ± 0.1 eV.

	**GO**		**$MoSe_2$/prGOAM**	
C 1s	*BE (eV)*	*%*	*BE (eV)*	*%*
C sp^2	284.6	54.5	284.5	62.3
C sp^3	285.6	10.9	285.5	10.8
C-O	286.4	13.8	286.4	15.6
Epoxide	287.0	8.9	287.0	2.9
C=O	288.0	4.7	288.0	4.3
O-C=O	288.8	7.2	289.0	4.2
C sp^x/CO_x	1.9		2.7	

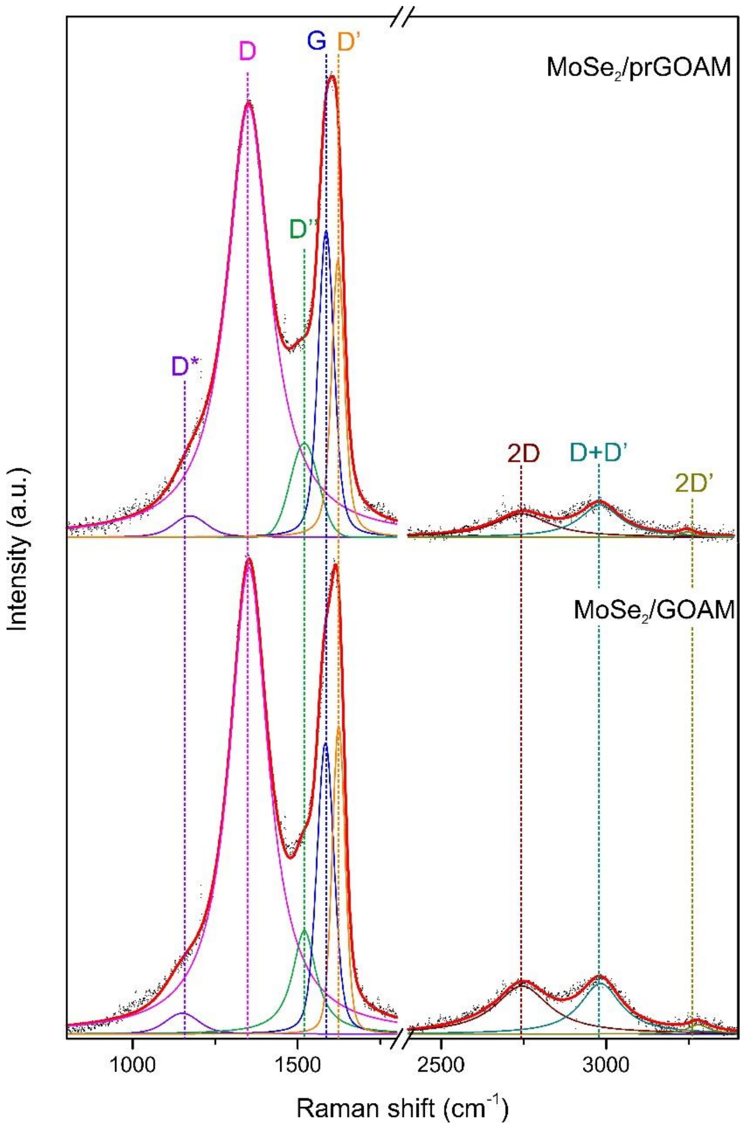

Figure S6. Deconvolution of the Raman spectrum of graphene oxide of $MoSe_2$/GOAM and $MoSe_2$/prGOAM reduced to 450 °C (right).

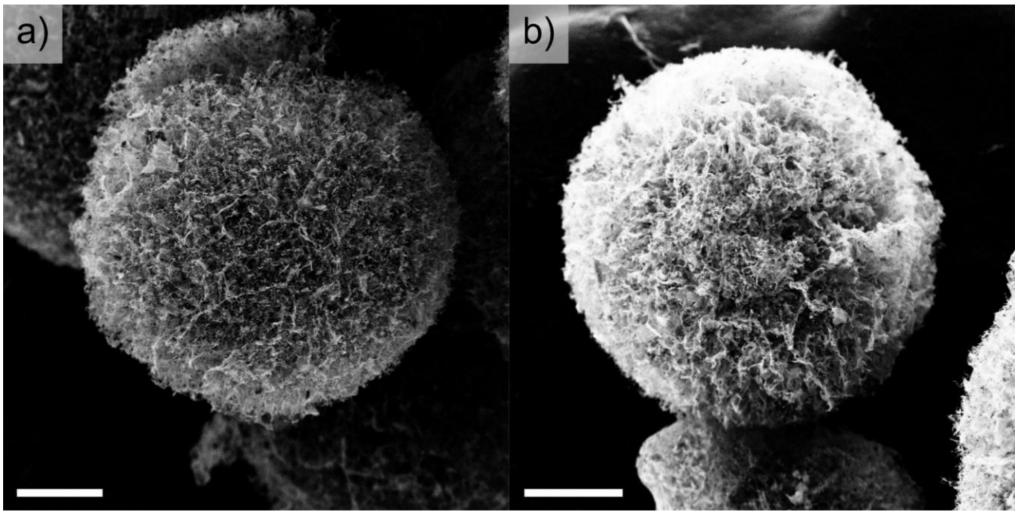

Figure S7. SEM images of $MoSe_2$/prGOAM samples obtained by reduction at 150 °C (left) and 900 °C (right). Scale bars are 100 µm.

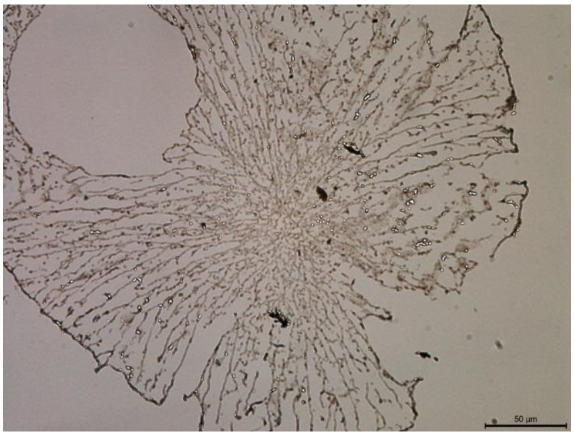

Figure S8. Optical microscope image (63x) of $MoSe_2$/prGOAM samples obtained by reduction at 450 °C with different scale bars.

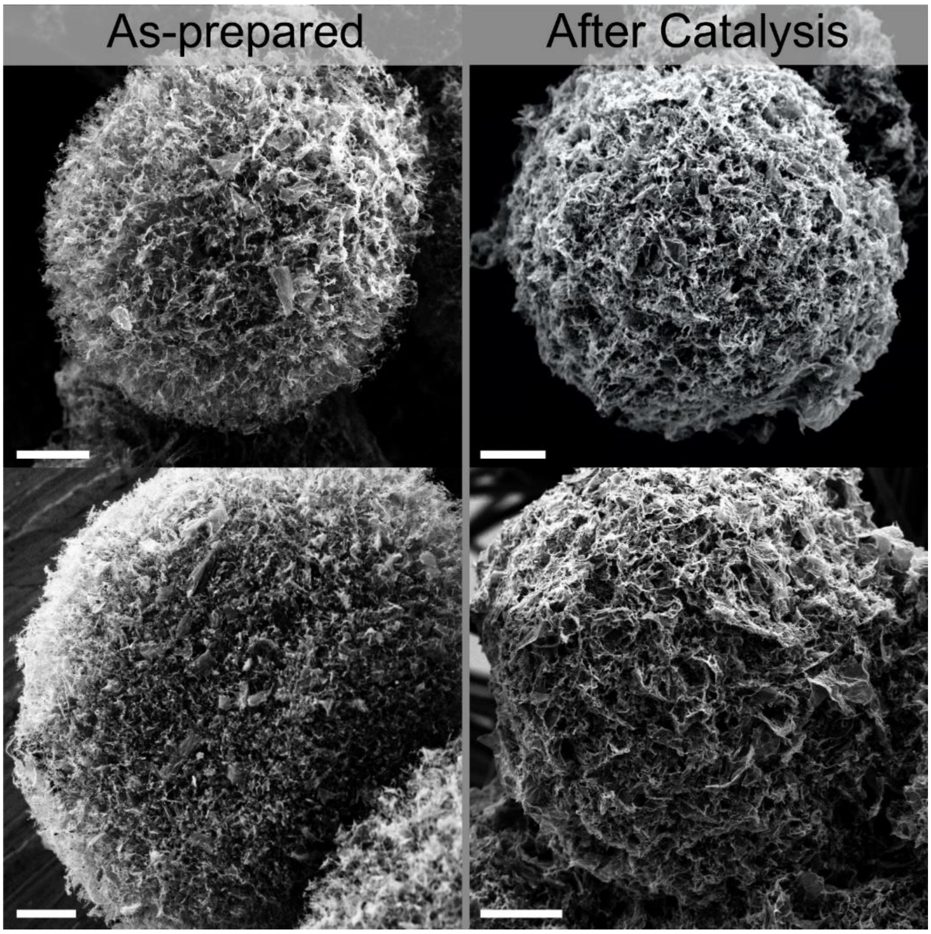

Figure S9. Comparison of the SEM images of the $MoSe_2$/prGOAM sample before and after the electrochemical tests. The reference sample is that obtained by heat treatment at 450 °C using a 5 mg/mL solution of $MoSe_2$. Scale bar are 50 μm.

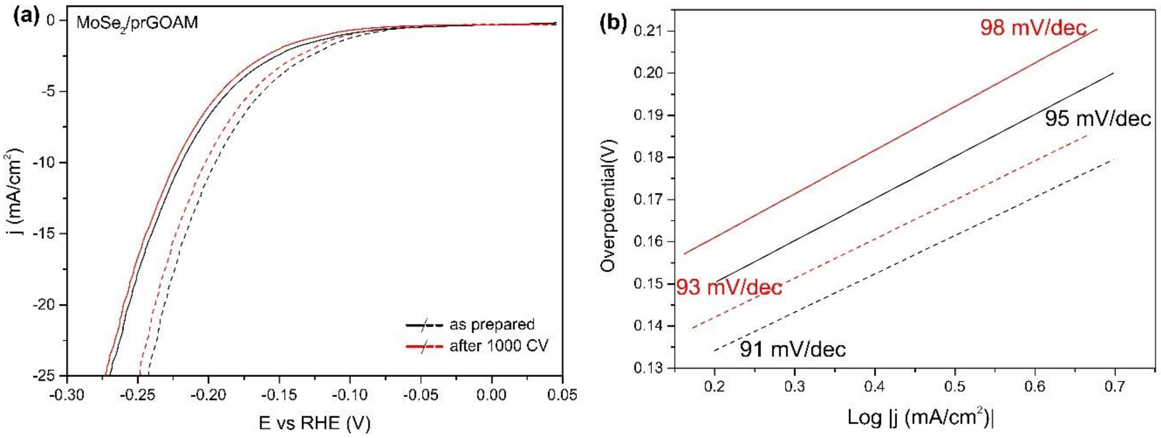

Figure S10. Polarization curves (a) and Tafel plots (b) for HER of as-prepared $MoSe_2$/prGOAM samples annealed at 450 °C (black) and after 1000 CVs under light exposure (red). Solid lines represent experiments in dark conditions, dashed lines the PEC-HER measurements performed under illumination.

Table S4. HER activity parameters of $MoSe_2$/prGOAM from data reported in Figure S10.

	**η_{10} (mV)**	**Tafel slope (mV/dec)**	**η_{10} LED (mV)**	**Tafel Slope LED (mV/dec)**	**$+j_{10}$ (mA/cm²)**
$MoSe_2$/prGOAM	-220 (5)	95 (1)	-194 (5)	91 (1)	6.6
$MoSe_2$/prGOAM (post EC)	-225 (5)	98 (2)	-203 (5)	93 (1)	5.3

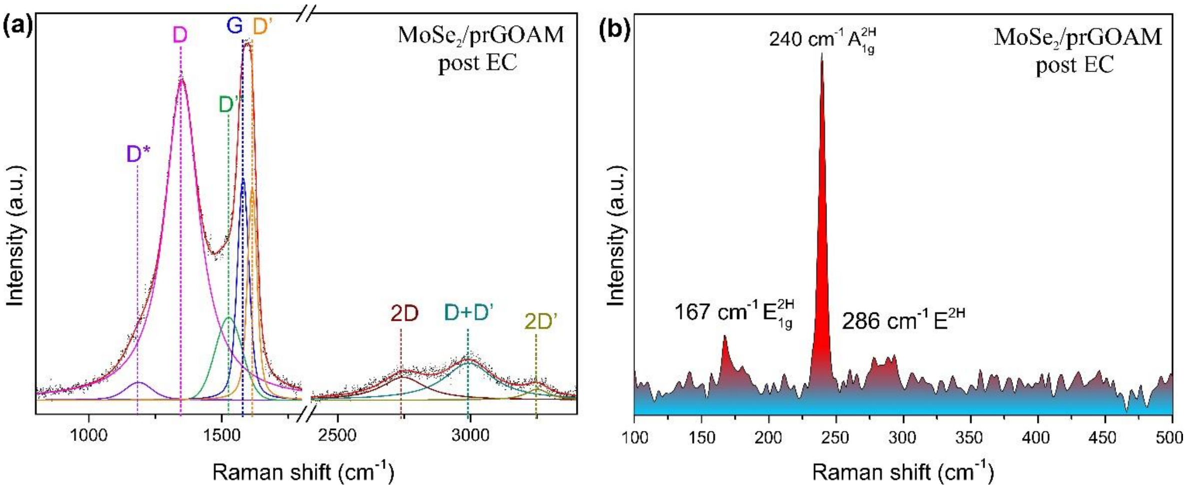

Figure S11. (a) Raman spectra of graphene oxide and (b) $MoSe_2$ of $MoSe_2$/prGOAM reduced to 450 °C after 1000 CVs under light exposure.

Table S5. Fitting parameters calculated for $MoSe_2$/GOAM and $MoSe_2$/prGOAM in the Raman spectra in Figure S6 and S11.

	ν (cm^{-1})									
	D*	D	D′	D″	G	2D	2D′	I_D/I_G	$I_{D'}/I_G$	$\nu_{2D} - 2\cdot\nu_D$
$MoSe_2$/GOAM	1150	1351	1624	1520	1584	2744	3178	1.58	1.05	42
$MoSe_2$/prGOAM	1174	1351	1622	1520	1585	2744	3190	1.41	0.90	42
$MoSe_2$/prGOAM (post EC)	1187	1351	1622	1522	1584	2775	3249	1.42	0.93	43

Extended characterization of exfoliated MoS_2, WS_2 and WSe_2

The TMDCs used were characterized by Raman and XPS comparing the exfoliated spectra with those of the bulk phase (Figures S8 for MoS_2, S10 for WS_2 and S12 for WSe_2). The bulk samples were used as an internal reference to characterize the related 2H phase. As seen with the $MoSe_2$, following the exfoliation process through the Li ions intercalation, there is a transition from the semiconductive phase to the metallic phase obtaining a system that presents both phases. In the case of MoS_2, the signals E_{2g} and A_{1g} of 2H phases ware identified at 382 and 407 cm^{-1} and are torn down during the transition to 1T. The latter instead is characterized by the presence of low Raman shift J_n signals, well clear in the spectrum of the exfoliated MoS_2 [1] (Figure S8). For the WS_2 the signals E_{2g} and A_{1g} of 2H phase appear at 350 and 416 cm^{-1}, respectively; following the exfoliation process, the migration of the A_{1g} signal to 407 cm^{-1} is observed, the abatement of the E_{2g} signal and the appearance of the J_1, A_{1g}, and J_3 peaks at 134, 278 and 318 cm^{-1}, respectively (Figure S10). These signals are compatible with 1T-WS_2 [2]. For the WSe_2, instead, the overlap of the E_{2g} and A_{1g} modes occurs, which results in a single peak at about 250 cm^{-1} both for the 2H and 1T phases (Figure S12) [3–6]. Through the study of XPS spectra (Figures S9 for MoS_2, S11 for WS_2 and S13 for WSe_2), the identification of the 1T phase is immediate. In the analysis of the signals of the exfoliated samples, for the presence of the metallic phase, it is necessary to take into account additional low-BE signals both for the transition metals (Mo 3d and W 4f) and for the chalcogens (S 2p and Se 3d). Similarly, to what described for the exfoliated $MoSe_2$, the 1T:2H ratio is calculated through the areas of the relative signals. For MoS_2 the doublets relative to 1T-Mo^{IV} and 1T-S^{2-} are displaced by the relative signals 2H of about 1 and 1.1 eV respectively. For WS_2 the distance from the 2H signals of 1T-W^{IV} and 1T-S^{2-} is about 0.9 and 0.6 eV, while for the WSe_2 the distance from the 2H signals of 1T-W^{IV} and 1T-Se^{2-} is about 0.5 eV for both signals [3]. In order to correctly interpolate the spectra of the W 4f region, it is necessary to take the signal into account W $5p_{3/2}$ (~ 37.4 eV) and, for the exfoliated samples, a doublet related to W^{V} species (34.7 eV), to consider partially oxidized species [7]. Full XPS data are reported in Tables S4-S9.

MoS2

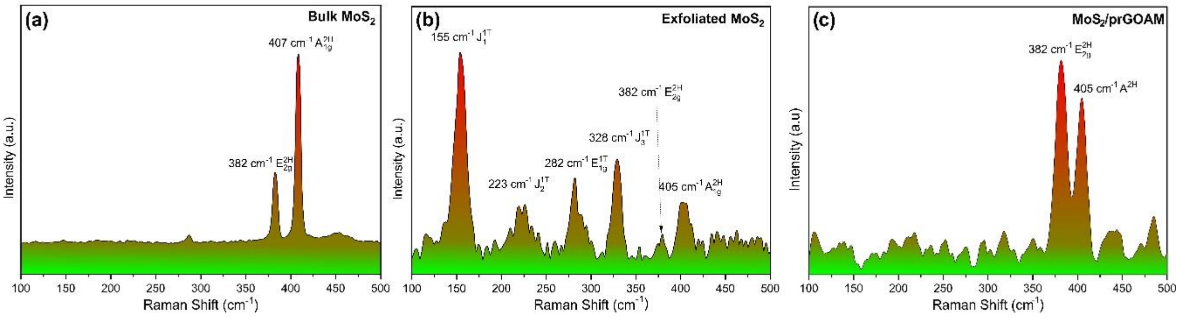

Figure S12. Raman spectra of bulk (a), exfoliated (b) and prGOAM hybrid (c) MoS_2 samples.

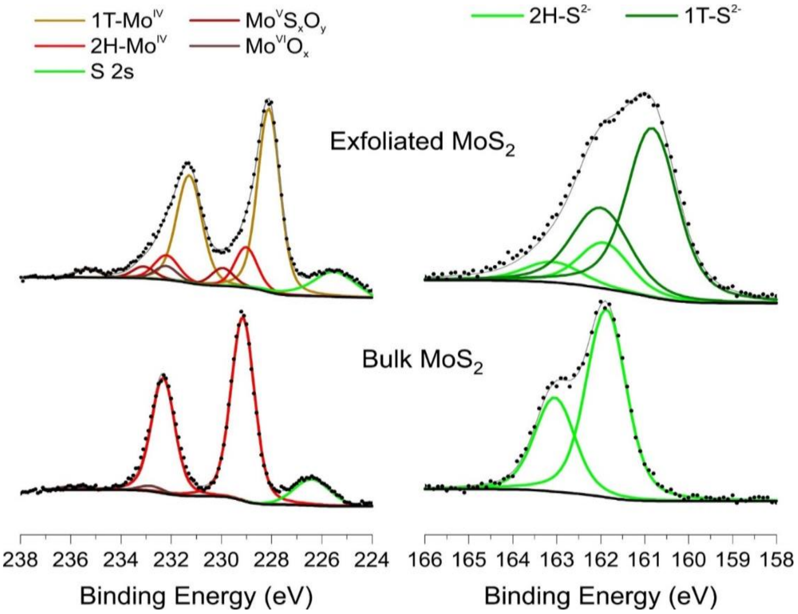

Figure S13. XPS spectra of Mo 3d (left) and S 2p (right) regions for exfoliated and bulk MoS_2 samples.

Table S6. BE values and composition of Mo 3d core level of MoS_2 samples. The reported BE values error is ± 0.1 eV. Percentage value of non-oxidized Mo and 1T:2H ratio of MoS_2 are reported as well.

	**Exfoliated MoS_2**		**Bulk MoS_2**	
Mo 3d	*BE (eV)*	*%*	*BE (eV)*	*%*
1T-Mo^{IV}	228.1	71.8	-	-
2H-Mo^{IV}	229.1	15.8	229.1	96.1
$Mo^VS_xO_y$	35.0	7.1		
$Mo^{VI}O_x$	232.3	5.3	232.3	3.9
1T:2H	82:18		0:100	
Mo^{IV}/Mo	88%		96%	

Table S7. BE values and composition of S 2p region of MoS_2 samples. S 2s BE values are reported as well. The error on BE values is ± 0.1 eV.

	**Exfoliated MoS_2**		**Bulk MoS_2**	
S 2p	*BE (eV)*	*%*	*BE (eV)*	*%*
1T-S^{2-}	160.8	21.0	-	-
2H-S^{2-}	161.9	79.0	161.9	100
S 2s	225.5	-	226.4	-

WS_2

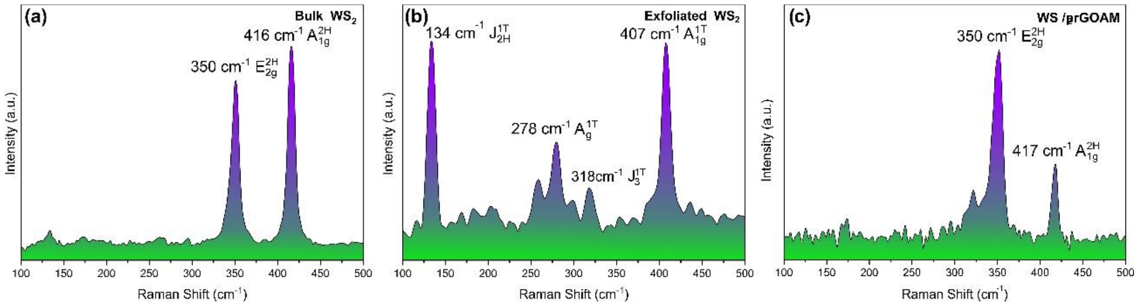

Figure S14. Raman spectra of bulk (a), exfoliated (b) and prGOAM hybrid (c) WS_2 samples.

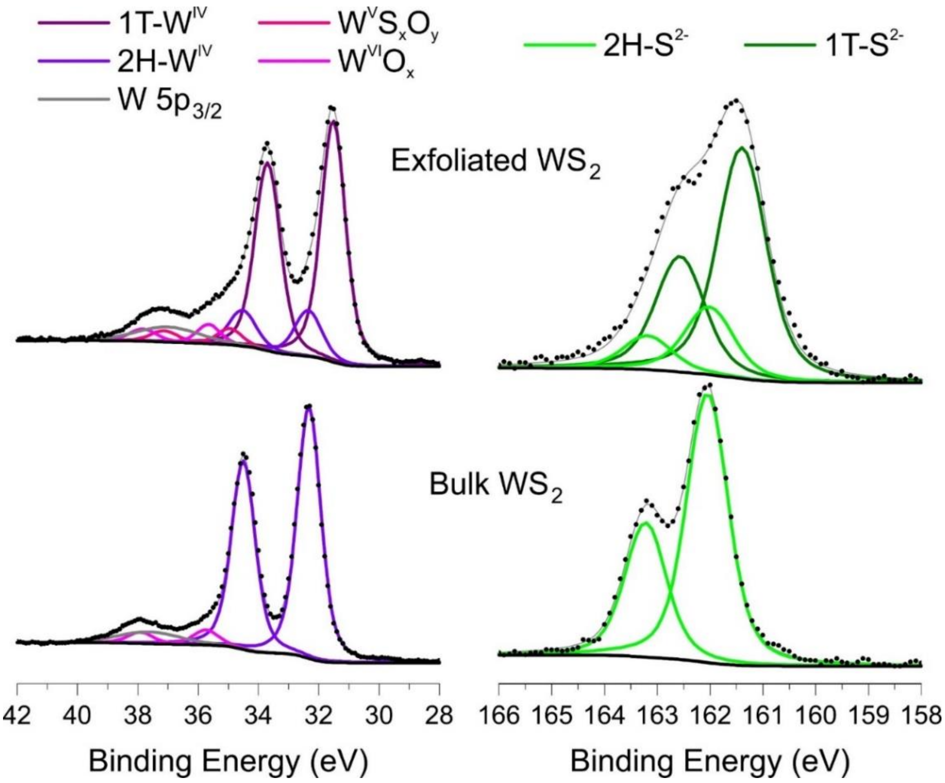

Figure S15. XPS spectra of W 4f (left) and S 2p (right) regions for exfoliated and bulk WS_2 samples.

Table S8. BE values and composition of W 4f core level of WS_2 samples. The reported BE values error is ± 0.1 eV. BE of W $5p_{3/2}$, percentage value of non-oxidized W and 1T:2H ratio of WS_2 are reported as well.

	**Exfoliated WS_2**		**Bulk WS_2**	
W 4f	*BE (eV)*	*%*	*BE (eV)*	*%*
1T-W^{IV}	31.5	74.2	-	
2H-W^{IV}	32.4	15.5	32.3	94.0
$W^{V}S_xO_y$	35.0	5.0	-	
$W^{VI}O_x$	35.7	5.3	35.7	6.0
W $5p_{3/2}$	37.0	-	37.6	-
1T:2H	83:17		0:100	
W^{IV}/W	90%		94%	

Table S9. BE values and composition of S 2p region of WS_2 materials. BE values error is ± 0.1 eV.

	**Exfoliated WS_2**		**Bulk WS_2**	
**S 2p**	*BE (eV)*	%	*BE (eV)*	%
**1T-S^{2-}**	161.4	79.3	-	-
**2H-S^{2-}**	162.0	19.7	162.1	100

WSe_2

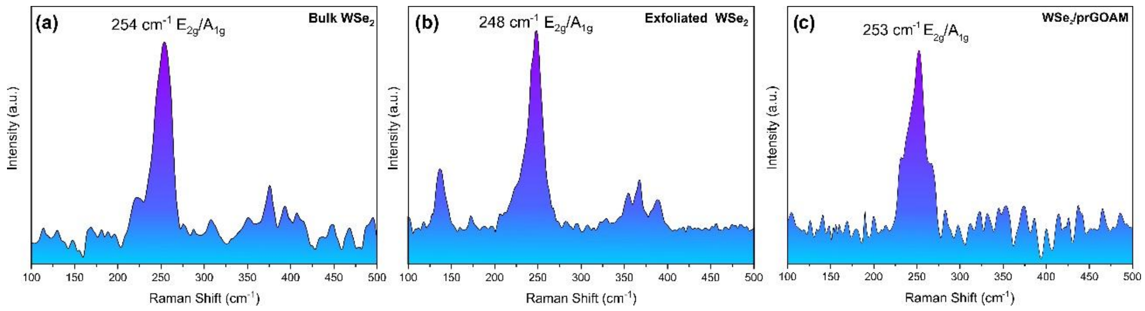

Figure S16. Raman spectra of bulk (a), exfoliated (b) and prGOAM hybrid (c) WSe_2 samples.

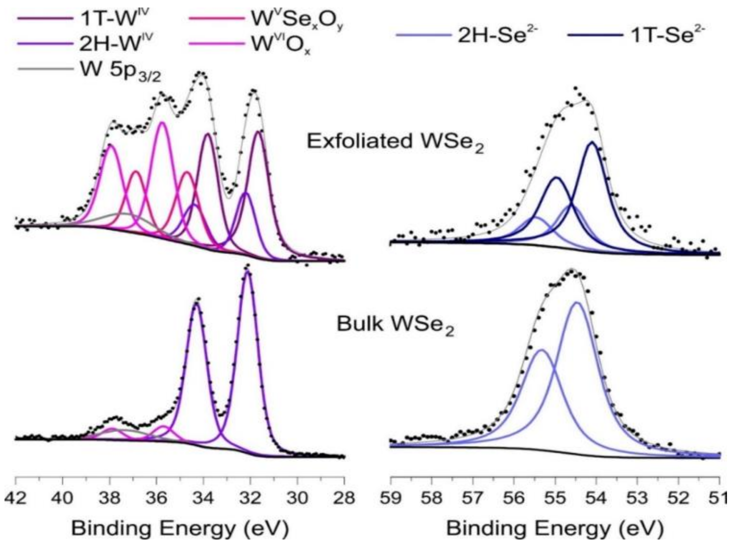

Figure S17. XPS spectra of W 4f (left) and Se 3d (right) regions for exfoliated and bulk WSe_2 samples.

Table S10. BE values and composition of W 4f core level of WSe_2 samples. The reported BE values error is ± 0.1 eV. BE of W $5p_{3/2}$, percentage value of non-oxidized W and 1T:2H ratio of WSe_2 are reported as well.

	**Exfoliated WSe_2**		**Bulk WSe_2**	
W 4f	*BE (eV)*	%	*BE (eV)*	%
1T-W^{IV}	31.5	36.5	-	-
2H-W^{IV}	32.2	14.4	32.1	87
$W^VS_xO_y$	34.7	19.3	-	-
$W^{VI}O_x$	35.8	29.8	35.7	13
W $5p_{3/2}$	37.2	-	37.5	-
1T:2H	72:28		0:100	
W^{IV}/W	52%		87%	

Table S11. BE values and composition of Se 3d region of WSe_2 materials. BE values error is ± 0.1 eV.

	**Exfoliated WSe_2**		**Bulk WSe_2**	
Se 3d	*BE (eV)*	*%*	*BE (eV)*	*%*
1T-Se^{2-}	54.1	71.4	-	-
2H-Se^{2-}	54.6	28.6	54.5	100

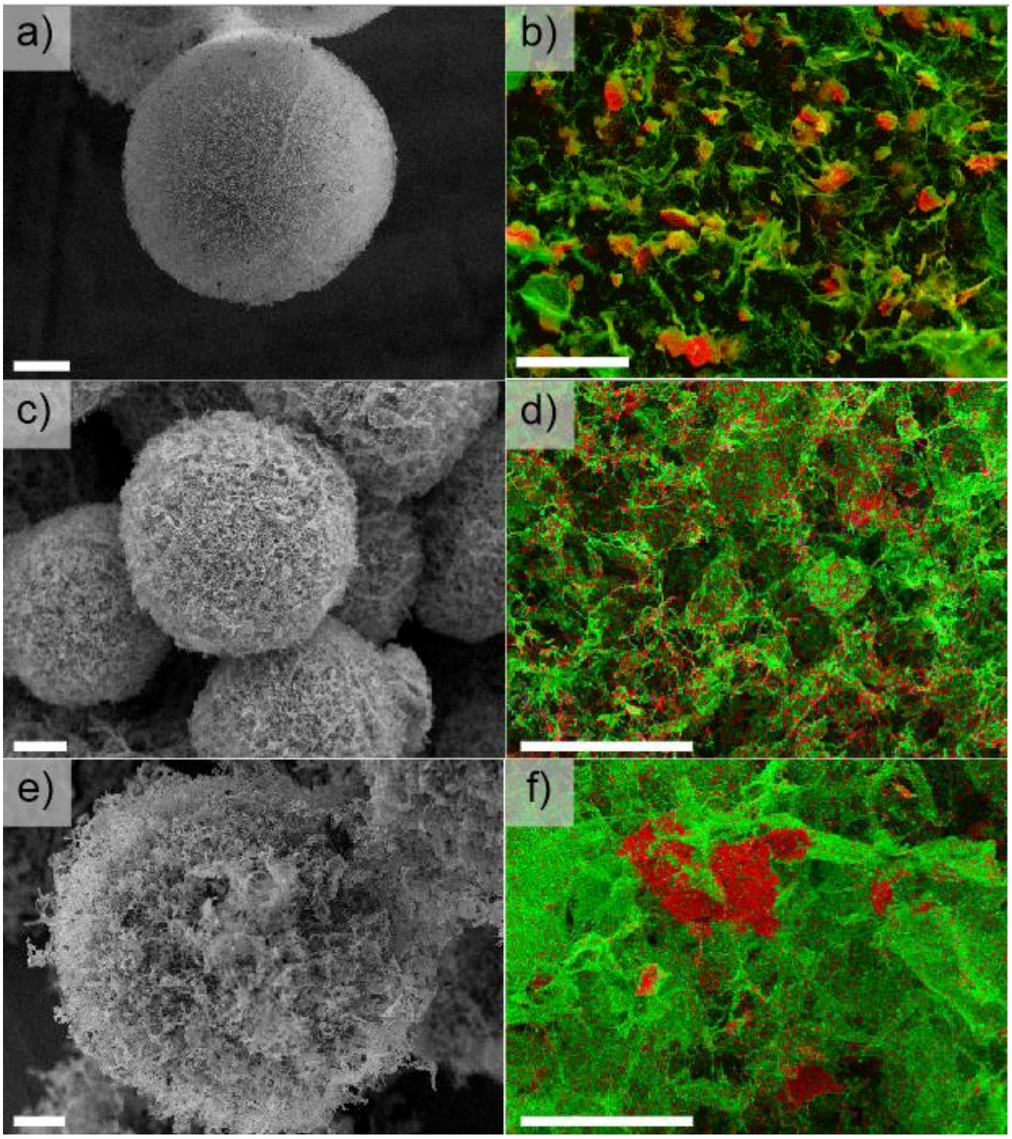

Figure S18. SEM images and related EDX maps of the samples a-b) MoS_2/prGOAM, c-d) WS_2/prGOAM and e-f) WSe_2/prGOAM. In EDX maps the green signal correspond to C, while red to Mo or W. Scale bars are 50 μm for SEM images and 20 μm for EDX maps.

References

1. Gupta, U.; Naidu, B.S.; Maitra, U.; Singh, A.; Shirodkar, S.N.; Waghmare, U. V; Rao, C.N.R. Characterization of few-layer 1T-MoSe 2 and its superior performance in the visible-light induced hydrogen evolution reaction. *APL Mater.* **2014**, *2*, 092802, doi:10.1063/1.4892976.
2. Liu, Z.; Li, N.; Su, C.; Zhao, H.; Xu, L.; Yin, Z.; Li, J.; Du, Y. Colloidal synthesis of 1T' phase dominated WS 2 towards endurable electrocatalysis. *Nano Energy* **2018**, *50*, 176–181, doi:10.1016/j.nanoen.2018.05.019.
3. Ambrosi, A.; Sofer, Z.; Pumera, M. 2H → 1T phase transition and hydrogen evolution activity of MoS 2 , MoSe 2 , WS 2 and WSe 2 strongly depends on the MX 2 composition. *Chem. Commun.* **2015**, *51*, 8450–8453, doi:10.1039/C5CC00803D.
4. Del Corro, E.; Botello-Méndez, A.; Gillet, Y.; Elias, A.L.; Terrones, H.; Feng, S.; Fantini, C.; Rhodes, D.; Pradhan, N.; Balicas, L.; et al. Atypical Exciton-Phonon Interactions in WS2 and WSe2 Monolayers Revealed by Resonance Raman Spectroscopy. *Nano Lett.* **2016**, *16*, 2363–2368, doi:10.1021/acs.nanolett.5b05096.
5. Ma, Y.; Liu, B.; Zhang, A.; Chen, L.; Fathi, M.; Shen, C.; Abbas, A.N.; Ge, M.; Mecklenburg, M.; Zhou, C. Reversible Semiconducting-to-Metallic Phase Transition in Chemical Vapor Deposition Grown Monolayer WSe2 and Applications for Devices. *ACS Nano* **2015**, *9*, 7383–7391, doi:10.1021/acsnano.5b02399.
6. Xu, Y.; Sheng, K.; Li, C.; Shi, G. Self-assembled graphene hydrogel via a one-step hydrothermal process. *ACS Nano* **2010**, *4*, 4324–4330.
7. Chen, T.Y.; Chang, Y.H.; Hsu, C.L.; Wei, K.H.; Chiang, C.Y.; Li, L.J. Comparative study on MoS2 and WS2 for electrocatalytic water splitting. *Int. J. Hydrogen Energy* 2013, *38*, 12302–12309.

© 2020 by the authors. Submitted for possible open access publication under the terms and conditions of the Creative Commons Attribution (CC BY) license (http://creativecommons.org/licenses/by/4.0/).
